# Safety and feasibility of liver resection including major hepatectomy for geriatric patients with hepatocellular carcinoma: a retrospective observational study

**DOI:** 10.1186/s12885-024-12514-0

**Published:** 2024-06-26

**Authors:** Hiroki Kanno, Kazuaki Hashimoto, Hisamune Sakai, Toshiro Ogata, Shogo Fukutomi, Masanori Akashi, Yuichi Goto, Takeshi Aoyagi, Masahiko Taniguchi, Toru Hisaka

**Affiliations:** 1 Department of Surgery, Kurume University School of Medicine, 67 Asahi-Machi, Kurume, 830-0011 Japan; 2 Department of Surgery, St. Mary’s Hospital, Kurume, Japan

**Keywords:** Hepatocellular carcinoma, Hepatectomy, Older adult patient, Postoperative complication, Prognosis

## Abstract

**Background:**

It is unclear whether hepatectomy, which ranges in invasiveness from partial to major hepatectomy, is safe and feasible for older adult patients. Therefore, we compared its postoperative complications and long-term outcomes between younger and older adult patients.

**Methods:**

Patients who underwent hepatectomies for hepatocellular carcinoma (*N* = 883) were evaluated. Patients were divided into two groups: aged < 75 years (*N *= 593) and ≥ 75 years (*N *= 290). Short-term outcomes and prognoses were compared between the groups in the entire cohort. The same analyses were performed for the major hepatectomy cohort.

**Results:**

In the entire cohort, no significant differences were found in complications between patients aged < 75 and ≥ 75 years, and the multivariate analysis did not reveal age as a prognostic factor for postoperative complications. However, overall survival was significantly worse in older patients, although no significant differences were noted in time to recurrence or cancer-specific survival. In the multivariate analyses of time to recurrence, overall survival, and cancer-specific survival, although older age was an independent poor prognostic factor for overall survival, it was not a prognostic factor for time to recurrence and cancer-specific survival. In the major hepatectomy subgroup, short- and long-term outcomes, including time to recurrence, overall survival, and cancer-specific survival, did not differ significantly between the age groups. In the multivariate analysis, age was not a significant prognostic factor for complications, time to recurrence, overall survival, or cancer-specific survival.

**Conclusion:**

Hepatectomy, including minor and major hepatectomy, may be safe and oncologically feasible options for selected older adult patients with hepatocellular carcinoma.

**Supplementary Information:**

The online version contains supplementary material available at 10.1186/s12885-024-12514-0.

## Background

The average life expectancy has increased owing to advances in medical techniques and innovations. This trend is particularly noticeable in Japan and Hong Kong, where the average life expectancy has been reported to be 81.6 years in men and 87.7 years in women, and 83.2 years in men and 87.9 years in women, respectively [1, 2]. Aging will become a future problem in other regions [3–5]. In general, geriatric patients tend to have more comorbidities, such as diabetes mellitus, cardiovascular, pulmonary, and renal disorders, than younger patients [6, 7]. Thus, older patients are often considered to have contraindications for surgery because of their age. However, various studies have reported that the short- and long-term postoperative outcomes of geriatric patients are comparable with those of younger patients [8–11].

Hepatectomy is a potentially curative treatment of choice for hepatocellular carcinoma (HCC), but it has higher morbidity and mortality rates than other abdominal surgeries [12–15]. Thus, surgical indications and methods of liver resection should be thoroughly discussed and strictly followed to avoid unnecessary complications and mortality. However, hepatectomy ranges in invasiveness from partial to major hepatectomy [16, 17]. We speculate that partial hepatectomy for geriatric patients is permissible because it is less invasive; however, whether major hepatectomies, such as bi- or trisectionectomy, are safe and feasible for older adult patients is unclear. In the future, more geriatric patients will require hepatectomies owing to improved life expectancy, regardless of the extent of liver resection.

Therefore, we compared the postoperative complications and long-term outcomes between younger and older adult patients with HCC who underwent hepatectomies. Additionally, we evaluated patients in the major hepatectomy cohort in a subgroup analysis.

## Methods

### Patients

The data of consecutive patients who underwent hepatectomies for HCC at Kurume University between January 2006 and December 2020 (*N *= 813) and St. Mary’s Hospital between January 2006 and December 2020 (*N *= 98) were retrospectively analyzed. The inclusion criteria were as follows: treatment-naïve HCC, initial hepatectomy, performance status ≤ 2, American Society of Anesthesiologists classification ≤ 3, and histopathological confirmation of HCC. Conversely, the exclusion criteria were as follows: curative resection not achieved (*N *= 13) and insufficient data (*N *= 15). In total, 883 patients were enrolled in this study. Patients were divided into two groups: < 75 years (*N *= 593) and ≥ 75 years (*N *= 290); the short-term outcomes and prognoses were compared between the groups in the entire cohort. Additionally, the same analyses were performed in the major hepatectomy cohort as a subgroup analysis.

The protocol for this research project has been approved by the Research Ethics Committee of Kurume University (no. 22294) and it conforms to the provisions of the Declaration of Helsinki. The need for informed consent was waived owing to the retrospective design of the study. We declare that we have ensured protection of the confidentiality of patient data.

### Data collection

Clinical and pathological data were obtained from the patient’s medical records. Blood samples and physical data were obtained within 1 week before surgery. Histopathological diagnoses were performed by at least two pathologists in accordance with the Liver Cancer Study Group of Japan guidelines.

### Treatment plan

In principle, we treated patients in accordance with the Japanese Clinical Practice Guidelines for Hepatocellular Carcinoma 2021 [18]. We diagnosed patients with underlying liver diseases, tumor markers, and imaging findings. A tumor biopsy was performed for patients with atypical imaging findings. We held weekly discussions with physicians, particularly for difficult cases. The Japanese Clinical Practice Guidelines for Hepatocellular Carcinoma indicate that the candidates for liver transplantation are limited to patients with Child–Pugh (CP) scores C. Therefore, none of the patients in the present study were eligible for transplantations.

### Preoperative therapy

Preoperative therapies, such as transcatheter arterial (chemo)embolizations and transhepatic arterial infusions, were performed in patients expected to be at high risk of recurrence. We conducted portal vein embolizations (PVEs) in patients with a risk of postoperative liver failure, such as those with a future remnant liver volume of < 40%. After confirming that the future liver remnant had increased by approximately 10% using CT volumetry according to PVE, radical resection was performed. Cases in which ascites appeared after PVE, portal venous pressure was high during PVE, and sufficient residual liver enlargement was not achieved after PVE were excluded. No patients underwent associated liver partition and portal vein ligation for staged hepatectomies.

### Surgical procedure

The surgical plan was carefully discussed and comprehensively chosen based on patients’ liver function (CP score, liver damage, platelet count, extent of cirrhosis, presence of esophageal varices, and splenomegaly), tumor factors (size, number, location, and distance to the major vessels), and comorbidities. Major hepatectomy was defined as hepatectomy with three or more Couinaud’s liver segments, and minor hepatectomy was defined as involving less than three segments according to the Brisbane 2000 terminology [19]. Surgical procedures are briefly described as follows. Liver mobilization was performed before liver transection if needed. The Pringle maneuver was conducted in principle. Parenchymal transection was performed using an ultrasonic surgical aspirator, ultrasonic coagulation dissector, or clamp crushing methods, according to the surgeon’s preferences.

### Postoperative follow-up

During postoperative follow-up, routine blood tests and tumor markers (alpha-fetoprotein [AFP] and protein induced by vitamin K absence or antagonist-II) were examined at least every 3 months. Additionally, imaging, such as ultrasonography or computed tomography, was performed every 3 months. If any recurrent findings were confirmed, gadolinium ethoxybenzyl diethylenetriamine pentaacetic acid-enhanced magnetic resonance imaging or contrast-enhanced ultrasonography was performed. Time to recurrence (TTR) was defined as the time from surgery to recurrence. Overall survival (OS) was defined as the time from surgery to death. Cancer-specific survival (CSS) was defined as the time from surgery to HCC-related death.

### Statistical analyses

Categorical variables are presented as numbers and percentages and were compared using Pearson’s chi-squared test. Continuous variables are presented as medians and ranges or interquartile ranges and were compared using the Wilcoxon rank-sum test. Covariates associated with postoperative complications and International Study Group of Liver Surgery (ISGLS) liver failure were evaluated using a logistic regression model for univariate and multivariate analyses, and odds ratios and 95% confidence intervals (95% CIs) were calculated. Statistically significant covariates found in univariate analysis were included in multivariate analysis. Survival curves were created using the Kaplan–Meier method and compared using the log-rank test. A Cox proportional hazards model was used for univariate and multivariate analyses to identify the risk factors for prognosis, and hazard ratios and 95% CIs were calculated. Statistically significant covariates in the univariate analysis were included in the multivariate analysis. All statistical analyses were performed using JMP Pro, version 15 (SAS Institute, Cary, NC, USA). Statistical significance was set at a *p*-value of < 0.05.

## Results

### Patient characteristics in the entire cohort

The clinical and pathological characteristics of patients aged < 75 and ≥ 75 years in the entire cohort are summarized in Table 1. Body mass index, American Society of. Anesthesiologists physical status (ASA-PS), underlying liver disease, total bilirubin level, serum albumin level, prothrombin time, serum AFP level, CP score, albumin-bilirubin (ALBI) score, ALBI grade, surgical method, operation time, estimated blood loss, and histological fibrosis grade were significantly different between the two groups (all *p *< 0.05). Less-invasive surgery may have been performed for the patients aged ≥ 75 years.

**Table 1:** Clinicopathological characteristics of patients aged < 75 and ≥ 75 years in the entire cohort

**Entire cohort**	**Patients aged < 75 years** (***N *= 593)**	**%**	**Patients aged ≥ 75 years** (***N *= 290)**	**%**	***P*-value**
**Age (median, IQR), years**	66 (60–71)		78 (76–81)		< .0001*
**Sex**					0.0585
Male	462	77.9%	209	72.1%	
Female	131	22.1%	81	27.9%	
**BMI (median, IQR)**	23.1 (21.1–25.8)		22.3 (20.2–24.9)		0.0002*
**ASA-PS**					< .0001*
1	39	6.6%	2	0.7%	
2	514	86.7%	260	89.7%	
3	40	6.7%	28	9.7%	
**Underlying liver disease**					< .0001*
HBV	146	24.6%	18	6.2%	
HCV	305	51.4%	176	60.7%	
NonBnonC	142	23.9%	96	33.1%	
**DM**					0.9265
No	386	65.1%	190	65.5%	
Yes	206	34.7%	100	34.5%	
Missing	1	0.2%	0	0.0%	
**T.bil (median, IQR), mg/dL**	0.75 (0.60–0.95)		0.70 (0.57–0.88)		0.0080*
**Alb (median, IQR), g/dL**	4.03 (3.74–4.32)		3.90 (3.60–4.20)		< .0001*
**PT (median, IQR), min**	92 (83–101)		96 (85–104)		0.0067*
**Plt (median, IQR), × 104/µL**	14.5 (10.9–18.7)		15.0 (12.2–18.6)		0.1149
**AFP (median, IQR), ng/mL**	12.3 (4.4–77.5)		6.9 (3.4–39.8)		0.0004*
**CP score**					0.0245*
A	566	95.4%	285	98.3%	
B	27	4.6%	5	1.7%	
**ALBI score (median, IQR)**	-2.7068 (-2.9306–2.4283)		-2.6156 (-2.8218–2.3407)		0.0006*
**ALBI grade**					0.0188*
1	364	61.4%	151	52.1%	
2	228	38.4%	137	47.2%	
3	1	0.2%	2	0.7%	
**MELD score (median, range**)	7 (6–12)		7 (6–13)		0.4397
**Operation approach**					0.2887
Open	444	74.9%	203	70.0%	
Laparoscopic	108	18.2%	61	21.0%	
Laparoscopic assisted	41	6.9%	26	9.0%	
**Operation method**					0.0489*
Minor	395	66.6%	212	73.1%	
Major	198	33.4%	78	26.9%	
**Operation time (median, IQR), min**	372 (291–465)		340 (260–420)		< .0001*
**Estimated blood loss (median, IQR), mL**	405 (164–810)		344 (114–656)		0.0080*
**Tumor diameter (median, IQR), mm**	27 (20–42)		30 (20–45)		0.2071
**Tumor number**					0.3644
Solitary	465	78.4%	235	81.0%	
Multiple	128	21.6%	55	19.0%	
**Macroscopic finding**					0.4800
Simple nodular or obscure	338	57.0%	173	62.9%	
Perinodular or multinodular	234	39.5%	100	36.4%	
Unclassifiable	21	3.5%	2	0.7%	
**Differentiation**					0.4768
Well and/or moderate	484	81.6%	232	80.0%	
Poor	87	14.7%	48	16.6%	
Unclassifiable or missing	22	3.7%	10	3.4%	
**Vascular invasion**					0.9823
No	250	42.2%	123	42.4%	
Yes	312	52.6%	153	52.8%	
Unclassifiable or missing	31	5.2%	14	4.8%	
**Inuyama fibrosis grade**					< .0001*
0–2	258	43.5%	178	61.4%	
3–4	320	54.0%	104	35.9%	
Missing	15	2.5%	8	2.8%	
**TNM classification**					
T					0.3161
1	103	17.4%	46	15.9%	
2	190	32.0%	95	32.8%	
3	192	32.4%	109	37.6%	
4	104	17.5%	40	13.8%	
Unclassifiable	4	0.7%	0	0.0%	
N					0.5219
0	589	99.3%	289	99.7%	
1	4	0.7%	1	0.3%	
M					0.2128
0	591	99.7%	287	99.0%	
1	2	0.3%	3	1.0%	
**Stage**					0.4414
1	104	17.5%	46	15.9%	
2	188	31.7%	94	32.4%	
3	191	32.2%	107	36.9%	
4	106	17.9%	43	14.8%	
Unclassifiable	4	0.7%	0	0.0%	

*AFP* Alpha-fetoprotein, *Alb* Albumin, *ALBI* Albumin-bilirubin, *ASA-PS* American Society of. Anesthesiologists physical status, *BMI* Body mass index, *CP* Child–Pugh, *DM* Diabetes mellitus, *HBV* Hepatitis B virus, *HCV* Hepatitis C virus, *IQR* Interquartile range, *MELD* Model for End-Stage Liver Disease, *Plt* Platelet, *PT* Prothrombin time, *T.bil* Total bilirubin

^*^Indicates that there is a significant difference

### Postoperative complications and ISGLS liver failure in the entire cohort

Clavien–Dindo (CD) postoperative complications, ISGLS liver failure, and in-hospital days were not significantly different between the two groups (Supplementary Table 1). ALBI grade, operation time, estimated blood loss, and fibrosis grade were predictive factors for complications in the univariate analysis (all *p *< 0.05). In the multivariate analysis, ALBI grade, operation time, and fibrosis grade were independent predictors of complications (all *p *< 0.05) (Supplementary Table 2).

For ISGLS liver failure, sex, ALBI grade, CP score, surgical method, operation time, and estimated blood loss were predictive factors in the univariate analysis (all *p *< 0.05). In the multivariate analysis, sex, ALBI grade, CP score, and estimated blood loss were independent predictors of ISGLS liver failure (all *p *< 0.05).

### Comparison of recurrence and survival between the two groups in the entire cohort

**Fig. 1:**
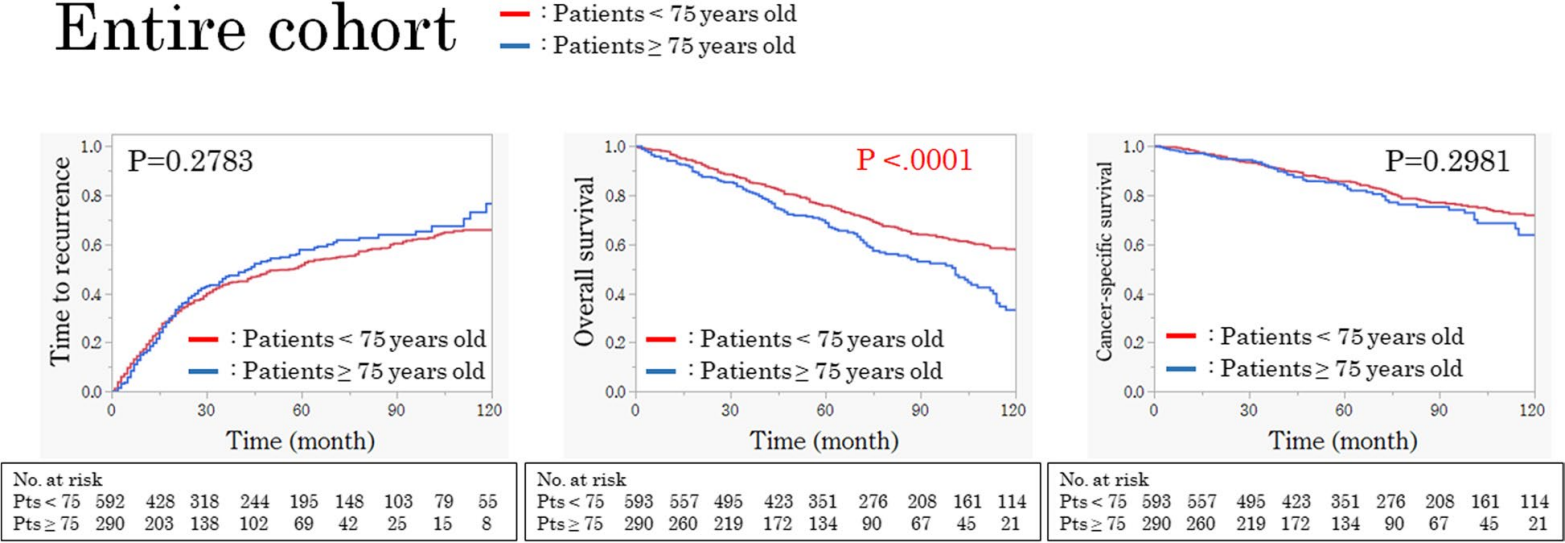
TTR, OS, and CSS curves of < 75 and ≥ 75-year-old patients in the entire cohort. Although the TTR and CSS were not significantly different between the groups, OS was significantly worse in patients aged ≥ 75 years. TTR, time to recurrence; OS, overall survival; CSS, cancer-specific survival

Although TTR and CSS were similar between the two groups, patients aged ≥ 75 years had significantly worse OS compared with patients aged < 75 years (*p* = 0.2783, *p* = 0.2981, and* p *< 0.0001, respectively) (Fig. 1).

### Univariate and multivariate analyses of TTR, OS, and CSS

The results of univariate and multivariate analyses of TTR, OS, and CSS are shown in Table 2.

**Table 2:** Univariate and multivariate analyses of TTR, OS, and CSS in the entire cohort

	**Univariate**		**Multivariate**	
***N *= 883**	**HR (95% CI)**	***P*-value**	**HR (95% CI)**	***P*-value**
**Time to recurrence**				
Sex (male vs. female)	1.1817 (0.9568–1.4595)	0.1211		
Age (< 75 vs. ≥ 75), years	0.9004 (0.7437–1.0901)	0.2822		
ASA-PS (1–2 vs. 3)	1.1696 (0.8292–1.6497)	0.3720		
Underlying liver disease (viral vs. non-viral)	1.0447 (0.8508–1.2828)	0.6761		
ALBI grade (1 vs. 2–3)	0.5078 (0.4245–0.6076)	< .0001*	0.5249 (0.4301–0.6406)	< .0001*
CP score (A vs. B)	0.6451 (0.4163–0.9995)	0.0498*	0.7714 (0.4795–1.2410)	0.2846
AFP (< 20 vs. ≥ 20), ng/mL	0.7552 (0.6301–0.9051)	0.0024*	0.8986 (0.7306–1.1051)	0.3109
Operation method (minor vs. major)	0.8419 (0.6963–1.0178)	0.0755		
Operation time (< 300 vs. ≥ 300), min	0.9837 (0.8097–1.1951)	0.8683		
Estimated blood loss (< 500 vs. ≥ 500), mL	0.8563 (0.7151–1.0254)	0.0916		
Tumor diameter (≤ 20 vs. > 20), mm	0.6610 (0.5416–0.8068)	< .0001*	0.6851 (0.5493–0.8544)	0.0008*
Tumor number (solitary vs. multiple)	0.6142 (0.4999–0.7547)	< .0001*	0.7040 (0.5643–0.8784)	0.0019*
Differentiation (well/mod vs. por)	0.6712 (0.5323–0.8465)	0.0008*	0.8927 (0.6874–1.1593)	0.3945
Vascular invasion (no vs. yes)	0.6286 (0.5212–0.7582)	< .0001*	0.6956 (0.5648–0.8568)	0.0006*
Histological fibrosis grade (F0-2 vs. F3-4)	0.8278 (0.6912–0.9915)	0.0401*	0.7596 (0.6239–0.9248)	0.0062*
Clavien-Dindo (0–2 vs. ≥ 3a)	0.8986 (0.7212–1.1198)	0.8986		
ISGLS liver failure (0-A vs. B-C)	0.6448 (0.5008–0.8302)	0.0007*	0.8655 (0.6580–1.1386)	0.3020
**Overall survival**				
Sex (male vs. female)	1.2885 (0.9882–1.6800)	0.0612		
Age (< 75 vs. ≥ 75), years	0.6209 (0.4962–0.7768)	< .0001*	0.5712 (0.4483–0.7280)	< .0001*
ASA-PS (1–2 vs. 3)	0.4746 (0.3323–0.6778)	< .0001*	0.4488 (0.3088–0.6523)	< .0001*
Underlying liver disease (viral vs. non-viral)	0.7705 (0.6045–0.9822)	0.0353*	0.8963 (0.6846–1.1735)	0.4259
ALBI grade (1 vs. 2–3)	0.4937 (0.3975–0.6131)	< .0001*	0.6010 (0.4736–0.7627)	< .0001*
CP score (A vs. B)	0.7422 (0.4421–1.2462)	0.2596		
AFP (< 20 vs. ≥ 20), ng/mL	0.6721 (0.5411–0.8348)	0.0003*	0.7522 (0.5824–0.9715)	0.0292*
Operation method (minor vs. major)	0.7597 (0.6071–0.9506)	0.0163*	0.8442 (0.6565–1.0856)	0.1870
Operation time (< 300 vs. ≥ 300), min	0.9194 (0.7187–1.1762)	0.5039		
Estimated blood loss (< 500 vs. ≥ 500), mL	0.6991 (0.5635–0.8674)	0.0011*	0.8072 (0.6332–1.0289)	0.0836
Tumor diameter (≤ 20 vs. > 20), mm	0.5941 (0.4634–0.7667)	< .0001*	0.7633 (0.5724–1.0180)	0.0660
Tumor number (solitary vs. multiple)	0.6465 (0.5061–0.8259)	0.0005*	0.7399 (0.5659–0.9674)	0.0277*
Differentiation (well/mod vs. others)	0.6109 (0.4651–0.8022)	0.0004*	0.8307 (0.6044–1.1419)	0.2532
Vascular invasion (no vs. yes)	0.6507 (0.5176–0.8180)	0.0002*	0.8164 (0.6326–1.0536)	0.1190
Histological fibrosis grade (F0-2 vs. F3-4)	0.9144 (0.7327–1.1411)	0.4283		
Clavien-Dindo (0–2 vs. ≥ 3a)	0.8791 (0.6786–1.1389)	0.3294		
ISGLS liver failure (0-A vs. B-C)	0.5706 (0.4283–0.7602)	0.0001*	0.7786 (0.5703–1.0629)	0.1150
**Cancer-specific survival**				
Sex (male vs. female)	1.2763 (0.8838–1.8432)	0.1931		
Age (< 75 vs. ≥ 75), years	0.8426 (0.6097–1.1644)	0.2994		
ASA-PS (1–2 vs. 3)	0.7320 (0.4066–1.3180)	0.2985		
Underlying liver disease (viral vs. non-viral)	1.1224 (0.7765–1.6225)	0.5391		
ALBI grade (1 vs. 2–3)	0.5148 (0.3813–0.6949)	< .0001*	0.5694 (0.4106–0.7895)	0.0007*
CP score (A vs. B)	0.8422 (0.3952–1.7948)	0.6564		
AFP (< 20 vs. ≥ 20), ng/mL	0.6352 (0.4705–0.8574)	0.0030*	0.9133 (0.6415–1.3002)	0.6147
Operation method (minor vs. major)	0.7428 (0.5446–1.0131)	0.0604		
Operation time (< 300 vs. ≥ 300), min	0.6827 (0.4726–0.9862)	0.0419*	0.9950 (0.6582–1.5040)	0.9808
Estimated blood loss (< 500 vs. ≥ 500), mL	0.6839 (0.5071–0.9223)	0.0128*	0.8844 (0.6201–1.2612)	0.4974
Tumor diameter (≤ 20 vs. > 20), mm	0.4276 (0.2898–0.6309)	< .0001*	0.5623 (0.3669–0.8619)	0.0082*
Tumor number (solitary vs. multiple)	0.6302 (0.4495–0.8834)	0.0074*	0.6963 (0.4827–1.0044)	0.0528
Differentiation (well/mod vs. others)	0.5017 (0.3502–0.7186)	0.0002*	0.6922 (0.4584–1.0451)	0.0801
Vascular invasion (no vs. yes)	0.4442 (0.3165–0.6234)	< .0001*	0.5915 (0.4069–0.8596)	0.0059*
Histological fibrosis grade (F0–2 vs. F3–4)	0.9987 (0.7353–0.3564)	0.9933		
Clavien–Dindo (0–2 vs. ≥ 3a)	0.8882 (0.6192–1.2740)	0.5194		
ISGLS liver failure (0–A vs. B–C)	0.4950 (0.3378–0.7255)	0.0003*	0.6783 (0.4478–1.0275)	0.0669

*AFP* Alpha-fetoprotein, *ALBI* Albumin-bilirubin, *ASA-PS* American Society of. Anesthesiologists physical status, *CI* Confidence interval, *CP* Child–Pugh, *HR* Hazard ratio, *ISGLS* International Study Group of Liver Surgery

^*^Indicates that there is a significant difference

Univariate analysis of TTR demonstrated that ALBI grade, CP score (A vs. B), AFP level (< 20 vs. ≥ 20 ng/mL), tumor diameter (≤ 20 vs. > 20 mm), tumor number (solitary vs. multiple), tumor differentiation (well/moderately defined vs. poorly defined), vascular invasion (- vs. +), histological fibrosis grade (F0-2 vs. F3-4), and ISGLS liver failure (0-A vs. B-C) were prognostic factors. In multivariate analysis, ALBI grade, tumor diameter (≤ 20 vs. > 20 mm), tumor number (solitary vs. multiple), vascular invasion (- vs. +), and histological fibrosis grade (F0-2 vs. F3-4) were independent prognostic factors.

Regarding OS, univariate analysis showed that age (< 75 vs. ≥ 75 years), ASA-PS (1–2 vs. 3), underlying liver disease (viral vs. non-viral), ALBI grade (1 vs. 2–3), AFP (< 20 vs. ≥ 20 ng/mL), surgical method (minor vs. major), estimated blood loss (< 500 vs. ≥ 500 mL), tumor diameter (≤ 20 vs. > 20 mm), tumor number (solitary vs. multiple), tumor differentiation (well/moderately differentiated vs. poorly differentiated), vascular invasion (- vs. +), and ISGLS liver failure (0-A vs. B-C) were prognostic factors. In multivariate analysis, age (< 75 vs. ≥ 75 years), ASA-PS (1–2 vs. 3), ALBI grade (1 vs. 2–3), AFP (< 20 vs. ≥ 20 ng/mL), and tumor number (solitary vs. multiple) were independent prognostic factors.

In terms of CSS, univariate analysis showed that ALBI grade (1 vs. 2–3), AFP level (< 20 vs. ≥ 20 ng/mL), operation time (< 300 vs. ≥ 300 min), estimated blood loss (< 500 vs. ≥ 500 mL), tumor diameter (≤ 20 vs. > 20 mm), tumor number (solitary vs. multiple), tumor differentiation (well/moderately differentiated vs. poorly differentiated), vascular invasion (- vs. +), and ISGLS liver failure (0-A vs. B-C) were prognostic factors. In multivariate analysis, ALBI grade (1 vs. 2–3), tumor diameter (≤ 20 vs. > 20 mm), and vascular invasion (- vs. +) were independent prognostic factors.

### Patient characteristics in the major hepatectomy cohort

The clinicopathological features of patients aged < 75 and ≥ 75 years in the major hepatectomy cohort are summarized in Table 3. Underlying liver disease, serum albumin level, serum AFP level, ALBI score, ALBI grade, operation time, and Inuyama fibrosis grade were significantly different between the two groups (all *p *< 0.05).

**Table 3:** Clinicopathological characteristics of patients aged < 75 and ≥ 75 years in the major hepatectomy cohort

**Major hepatectomy**	**Patients aged < 75 years** (***N* = 198)**	**%**	**Patients aged ≥ 75 years** (***N* = 78)**	**%**	***P*-value**
**Age (median, IQR), years**	67 (60.8–70.3)		78 (76–80)		< .0001*
**Sex**					0.9941
Male	160	80.8%	63	80.8%	
Female	38	19.2%	15	19.2%	
**BMI (median, IQR)**	23.1 (21.4–25.5)		22.4 (19.9–25.2)		0.0593
**ASA-PS**					0.1187
1	14	7.0%	1	1.3%	
2	174	87.9%	72	92.3%	
3	10	5.1%	5	6.4%	
**Underlying liver disease**					0.0012*
HBV	55	27.8%	7	9.0%	
HCV	86	43.4%	39	50.0%	
Non B non C	57	28.8%	32	41.0%	
**DM**					0.5646
No	131	66.2%	49	62.8%	
Yes	66	33.3%	29	37.2%	
Missing	1	0.5%	0	0.0%	
**T.bil (median, IQR), mg/dL**	0.70 (0.56–0.88)		0.70 (0.55–0.89)		0.6852
**Alb (median, IQR), g/dL**	4.04 (3.76–4.32)		3.88 (3.60–4.20)		0.0062*
**PT (median, IQR), min**	94 (86–103)		98 (87.8–108)		0.0643
**Plt (median, IQR), × 10**^**4**^**/µL**	16.8 (13.1–20.8)		16.5 (14.1–19.5)		0.8414
**AFP (median, IQR), ng/mL**	22.2 (4.6–449.3)		5.7 (3.2–69.9)		0.0011*
**CP score**					0.8334
A	192	97.0%	76	97.4%	
B	6	3.0%	2	2.6%	
**ALBI score (median, IQR)**	-2.5567 (-2.7550–2.2523)		-2.4505 (-2.6061–2.1884)		0.0162*
**ALBI grade**					0.0362*
1	86	43.4%	21	26.9%	
2	110	55.6%	56	71.8%	
3	2	1.0%	1	1.3%	
**MELD score (median, range)**	7 (6–20)		7 (6–13)		0.6437
**Preoperative therapy**					0.2019
Yes	28	14.1%	16	20.5%	
No	170	85.9%	62	79.5%	
**Portal vein embolization**					0.1906
Yes	48	24.2%	25	32.1%	
No	150	75.8%	53	67.9%	
**Operation approach**					0.1743
Open	175	88.4%	65	83.3%	
Laparoscopic	5	2.5%	6	7.7%	
Laparoscopic assisted	18	9.1%	7	9.0%	
**Type of hepatectomy**					0.6178
Right hepatectomy	59	29.8%	27	27.0%	
Extended right hepatectomy	11	5.6%	6	6.0%	
Right tri-sectionectomy	5	2.5%	1	1.0%	
Left hepatectomy	47	23.7%	15	15.0%	
Extended left hepatectomy	30	15.2%	12	12.0%	
Left tri-sectionectomy	4	2.0%	0	0.0%	
Central hepatectomy	42	21.2%	17	17.0%	
**Operation time (median, IQR), min**	431.5 (356.8–529.8)		401 (337.8–482.3)		0.0164*
**Estimated blood loss (median, IQR), mL**	573 (292.5–1025)		510.5 (274.5–857.5)		0.4052
**Tumor diameter (median, IQR), mm**	41.5 (26.8–68.5)		44.5 (29.8–74.3)		0.6259
**Tumor number**					0.6881
Solitary	153	77.3%	62	79.5%	
Multiple	45	22.7%	16	20.5%	
**Macroscopic finding**					0.2517
Simple nodular or obscure	94	47.5%	43	55.1%	
Perinodular or multinodular	91	46.0%	26	33.3%	
Unclassifiable	13	6.6%	9	11.5%	
**Differentiation**					0.9157
Well and/or mod	147	74.2%	58	74.4%	
Poor	42	21.2%	16	20.5%	
Unclassifiable or missing	9	4.5%	4	5.1%	
**Vascular invasion**					0.7092
No	55	27.8%	24	30.8%	
Yes	128	64.6%	50	64.1%	
Unclassifiable or missing	15	7.6%	4	5.1%	
**Inuyama fibrosis grade**					0.0138*
0–2	119	60.1%	60	76.9%	
3–4	71	35.9%	17	21.8%	
Missing	8	4.0%	1	1.3%	
**TNM classification**					
T					0.9351
1	9	4.6%	4	5.1%	
2	51	25.8%	21	26.9%	
3	89	44.9%	32	41.1%	
4	48	24.2%	21	26.9%	
Unclassifiable	1	0.5%	0	0.0%	
N					0.1567
0	195	98.5%	78	######	
1	3	1.5%	0	0.0%	
M					0.1357
0	196	99.0%	75	96.2%	
1	2	1.0%	3	3.8%	
**Stage**					0.7694
1	10	5.1%	4	5.1%	
2	49	24.7%	21	26.9%	
3	89	45.0%	30	38.5%	
4	49	24.7%	23	29.5%	
Unclassifiable	1	0.5%	0	0.0%	

*AFP* Alpha-fetoprotein, *Alb* Albumin, *ALBI* Albumin-bilirubin, *ASA-PS* American Society of. Anesthesiologists physical status, *BMI* Body mass index, *CP* Child–Pugh, *DM* Diabetes mellitus, *HBV* Hepatitis B virus, *HCV* Hepatitis C virus, *IQR* Interquartile range, *MELD* Model for End-Stage Liver Disease, *Plt* Platelet, *PT* Prothrombin time, *T.bil* Total bilirubin

^*^Indicates that there is a significant difference

### Postoperative complications and ISGLS liver failure in the major hepatectomy cohort

CD postoperative complications, ISGLS liver failure, and in-hospital days were not significantly different between the two age groups (Supplementary Table 3). Univariate and multivariate analyses of the CD postoperative complications and ISGLS liver failure are shown in Supplementary Table 4. No significant differences were found between the groups for postoperative complications.

For ISGLS liver failure, estimated blood loss (< 500 vs. ≥ 500 mL) was a predictive factor in the univariate and multivariate analyses.

### Comparison of recurrence and survival between the two age groups in the major hepatectomy cohort

**Fig. 2:**
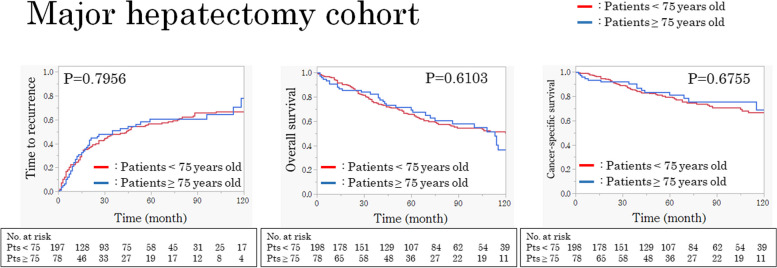
TTR, OS, and CSS curves of < 75 and ≥ 75-year-old patients in the major hepatectomy cohort. No significant differences were observed in all the curves between the groups. TTR, time to recurrence; OS, overall survival; CSS, cancer-specific survival

TTR, OS, and CSS were not significantly different between the two groups (*p *= 0.7956, *p *= 0.6103, and *p *= 0.6755, respectively; Fig. 2).

### Univariate and multivariate analyses of TTR, OS, and CSS in the major hepatectomy cohort

The results of the univariate and multivariate analyses of TTR, OS, and CSS are shown in Table 4. Univariate analysis of TTR demonstrated that sex (male vs. female), ALBI grade (1 vs. 2–3), tumor diameter (≤ 20 vs. > 20 mm), tumor number (solitary vs. multiple), tumor differentiation (well/moderately differentiated vs. poorly differentiated), vascular invasion (- vs. +), and ISGLS liver failure (0-A vs. B-C) were prognostic factors. In multivariate analysis, tumor number (solitary vs. multiple) and ISGLS liver failure (0-A vs. B-C) were independent prognostic factors.

**Table 4:** Univariate and multivariate analyses of TTR, OS, and CSS in the major hepatectomy cohort

	**Univariate**		**Multivariate**	
***N*= 276**	**HR (95% CI)**	***P*-value**	**HR (95% CI)**	***P*-value**
**Time to recurrence**				
Sex (male vs. female)	1.5556 (1.0072–2.4027)	0.0463*	1.2347 (0.7797–1.9550)	0.3687
Age (< 75 vs. ≥ 75), years	0.9554 (0.6741–1.3541)	0.7975		
ASA-PS (1–2 vs. 3)	1.2897 (0.6330–2.6276)	0.4835		
Underlying liver disease (viral vs. non-viral)	1.0758 (0.7590–1.5250)	0.6813		
ALBI grade (1 vs. 2–3)	0.7006 (0.5060–0.9700)	0.0321*	0.7088 (0.5004–1.0039)	0.0526
CP score (A vs. B)	0.4932 (0.2308–1.0537)	0.0680		
AFP (< 20 vs. ≥ 20), ng/mL	0.7316 (0.5345–1.0014)	0.0511		
Operation time (< 300 vs. ≥ 300), min	0.8097 (0.4826–1.3586)	0.4241		
Estimated blood loss (< 500 vs. ≥ 500), mL	0.7677 (0.5601–1.0524)	0.1005		
Tumor diameter (≤ 20 vs. > 20), mm	0.5772 (0.3484–0.9563)	0.0329*	0.6893 (0.3904–1.2169)	0.1995
Tumor number (solitary vs. multiple)	0.6100 (0.4282–0.8690)	0.0062*	0.6136 (0.4257–0.8846)	0.0089*
Differentiation (well/mod vs. others)	0.6666 (0.4612–0.9633)	0.0309*	0.8336 (0.5582–1.2451)	0.3740
Vascular invasion (no vs. yes)	0.6201 (0.4324–0.8894)	0.0094*	0.8048 (0.5484–1.1811)	0.2673
Histological fibrosis grade (F0–2 vs. F3–4)	0.9964 (0.7169–1.3848)	0.9828		
Clavien–Dindo (0–2 vs. ≥ 3a)	0.9421 (0.6295–1.4100)	0.7720		
ISGLS liver failure (0–A vs. B–C)	0.4965 (0.3403–0.7243)	0.0003*	0.6257 (0.4156–0.9421)	0.0247*
**Overall survival**				
Sex (male vs. female)	1.2048 (0.7584–1.9141)	0.4300		
Age (< 75 vs. ≥ 75), years	0.9010 (0.6026–1.3471)	0.6114		
ASA-PS (1–2 vs. 3)	0.8837 (0.4114–1.8982)	0.7512		
Underlying liver disease (viral vs. non-viral)	0.6877 (0.4710–1.0040)	0.0525		
ALBI grade (1 vs. 2–3)	0.6548 (0.4478–0.9575)	0.0290*	0.7786 (0.5276–1.1490)	0.2075
CP score (A vs. B)	1.3062 (0.4150–4.1109)	0.6480		
AFP (< 20 vs. ≥ 20), ng/mL	0.6382 (0.4454–0.9145)	0.0144*	0.6475 (0.4480–0.9358)	0.0207*
Operation time (< 300 vs. ≥ 300), min	0.7733 (0.4043–1.4790)	0.4371		
Estimated blood loss (< 500 vs. ≥ 500), mL	0.5361 (0.3657–0.7858)	0.0014*	0.6040 (0.4058–0.8989)	0.0129*
Tumor diameter (≤ 20 vs. > 20), mm	0.4982 (0.2675–0.9279)	0.0281*	0.6036 (0.3204–1.1372)	0.1183
Tumor number (solitary vs. multiple)	0.5650 (0.3821–0.8353)	0.0042*	0.5872 (0.3918–0.8801)	0.0099*
Differentiation (well/mod vs. others)	0.7175 (0.4682–1.0995)	0.1274		
Vascular invasion (no vs. yes)	0.6908 (0.4579–1.0421)	0.0779		
Histological fibrosis grade (F0–2 vs. F3–4)	1.0252 (0.6996–1.5025)	0.8983		
Clavien–Dindo (0–2 vs. ≥ 3a)	0.8066 (0.5250–1.2391)	0.3264		
ISGLS liver failure (0–A vs. B–C)	0.5769 (0.3750–0.8874)	0.0123*	0.7061 (0.4492–1.1088)	0.1307
**Cancer-specific survival**				
Sex (male vs. female)	1.4061 (0.7151–2.7648)	0.3232		
Age (< 75 vs. ≥ 75), years	1.1317 (0.6335–2.0215)	0.6761		
ASA-PS (1–2 vs. 3)	1.6334 (0.3990–6.6864)	0.4950		
Underlying liver disease (viral vs. non-viral)	1.0480 (0.5998–1.6673)	0.8691		
ALBI grade (1 vs. 2–3)	0.8640 (0.5214–1.4319)	0.5707		
CP score (A vs. B)	1.0005 (0.2445–4.0938)	0.9994		
AFP (< 20 vs. ≥ 20), ng/mL	0.5542 (0.3363–0.9132)	0.0205*	0.6992 (0.3833–1.2753)	0.2432
Operation time (< 300 vs. ≥ 300), min	1.0303 (0.4693–2.2617)	0.9407		
Estimated blood loss (< 500 vs. ≥ 500), mL	0.8422 (0.5113–1.3874)	0.5002		
Tumor diameter (≤ 20 vs. > 20), mm	0.3436 (0.1246–0.9475)	0.0390*	0.2545 (0.0603–1.0744)	0.0626
Tumor number (solitary vs. multiple)	0.5704 (0.3329–0.9775)	0.0411*	0.5865 (0.3296–1.0436)	0.0696
Differentiation (well/mod vs. por)	0.5328 (0.3060–0.9279)	0.0261*	0.7827 (0.4158–1.4735)	0.4478
Vascular invasion (no vs. yes)	0.3704 (0.1869–0.7340)	0.0044*	0.4799 (0.2263–1.0178)	0.0556
Histological fibrosis grade (F0–2 vs. F3–4)	1.2030 (0.6980–2.0734)	0.5056		
Clavien–Dindo (0–2 vs. ≥ 3a)	0.7663 (0.4290–1.3689)	0.3685		
ISGLS liver failure (0–A vs. B–C)	0.4408 (0.2513–0.7733)	0.0043*	0.5701 (0.3072–1.0581)	0.0749

*AFP* Alpha-fetoprotein, *ALBI* Albumin-bilirubin, *ASA-PS* American Society of. Anesthesiologists physical status, *CI* Confidence interval, *CP* Child–Pugh, *HR* Hazard ratio, *ISGLS* International Study Group of Liver Surgery

^*^Indicates that there is a significant difference

For OS, univariate analysis showed that ALBI grade (1 vs. 2–3), AFP (< 20 vs. ≥ 20 ng/mL), estimated blood loss (< 500 vs. ≥ 500 mL), tumor diameter (≤ 20 vs. > 20 mm), tumor number (solitary vs. multiple), and ISGLS liver failure (0-A vs. B-C) were prognostic factors. On multivariate analysis, AFP (< 20 vs. ≥ 20 ng/mL), estimated blood loss (< 500 vs. ≥ 500 mL), and tumor number (solitary vs. multiple) were independent prognostic factors.

Regarding CSS, univariate analysis showed that AFP (< 20 vs. ≥ 20 ng/mL), tumor diameter (≤ 20 vs. > 20 mm), tumor number (solitary vs. multiple), tumor differentiation (well/moderately differentiated vs. poorly differentiated), vascular invasion (- vs. +), and ISGLS liver failure (0-A vs. B-C) were prognostic factors. In multivariate analysis, there were no significant differences between the groups.

## Discussion

A nationwide study from the Netherlands has demonstrated that the incidence of liver-specific complications was not different between patients aged < 70 and ≥ 70 years; however, other complications, such as cardiac complications, pneumonia, and thromboembolism, occurred more frequently in older patients [20]. Similarly, a Japanese study based on a national clinical database has revealed that the occurrence of surgery-related complications did not differ between younger and older patients [21]. Additionally, Shimada et al. have shown that there were no significant differences among the three age groups (65 ≥ vs. 65–80 vs. ≥ 80 years) regarding postoperative complications [9]. These studies suggest that age alone is not a contraindication for surgery owing to postoperative complications. However, liver resection ranges in invasiveness from partial hepatectomy for small tumors at the liver edge to major hepatectomy for tumors located in the center of the liver or tumors attached to major vessels. In the present study, we evaluated the major hepatectomy cohort in a subgroup analysis and found no significant difference in postoperative complications between the two age groups. Therefore, not only minor liver resection but also major hepatectomy could be safely performed in geriatric patients.

The etiology of the underlying liver disease differed between the groups both in the entire and major hepatectomy cohorts (Tables 1 and 3). Tanaka et al. estimated that 1.7–2.2 million patients had chronic hepatitis C virus (HCV) in 2000 in Japan. The number of patients gradually reduced to 0.88–1.30 million in 2015 and are expected to be 0.21–0.48 million in 2030. However, the number of patients with hepatitis B virus (HBV) was estimated at 1.3–1.5 million in 2000 and then slowly reduced to 1.03–1.19 million in 2015. In 2030, it will be expected to reach 0.71–0.83 million [22]. Compared with that of HBV, the incidence of HCV has radically decreased; this may be owing to the use of direct-acting antivirals. It can achieve sustained viral response almost completely. The incidence of non-B non-C hepatitis-related HCC has been increasing, and the age of these patients is higher than that of those with HCV- and HBV-related HCC. These data are almost in line with those of the present study [23]. Therefore, our data may be a typical example of the trend of HCC occurrence in Japan.

Although a significant difference was found in OS between the two age groups in the entire cohort, TTR and CSS were not significantly different. Additionally, age was a prognostic factor for OS in the multivariate analysis. In contrast, in the major hepatectomy cohort, no significant differences were noted in TTR, OS, or CSS. This finding is partially consistent with the results of other studies. In a study by Liu et al., which included 1004 patients with HCC who underwent both minor and major hepatectomy, patients aged ≥ 75 years had significantly worse OS, whereas recurrence-free survival was similar between the age groups [24]. Chen et al. have reported that OS and recurrence-free survival after major hepatectomy for large HCC were comparable between patients aged ≥ 65 years (*N* = 92) and < 65 years *N *= 738) [25]. The worse OS outcomes for elderly patients in the entire cohort may be attributed to comorbidity. Patients aged ≥ 75 years have a higher prevalence of comorbidities than patients aged < 75 years: cardiac, 10.3% vs. 3.9%; pulmonary, 2.1% vs. 1.9%; and renal, 3.1% vs. 1.5% (Supplementary Table 5). In contrast, patients aged ≥ 75 years in the major hepatectomy cohort had a lower prevalence of comorbidities: cardiac, 3.1%; pulmonary, 0%; and renal, 1.0%. Overall, only patients with few comorbidities may be selected as candidates for major hepatectomy.

Regarding patient characteristics in the entire cohort, minor hepatectomy, shorter operation time, and less blood loss were observed in the older group, which is consistent with the results of other studies [8, 21]. Similar findings were observed in the major hepatectomy cohort. These findings suggest that there are stricter criteria for older patients and that challenging cases may potentially be avoided by surgeons. At present, although there are various tools to assess immunonutritional status, such as the prognostic nutritional index, Controlling Nutritional Status score, and Glasgow prognostic score, as well as examining sarcopenia and the area of the iliopsoas muscle at the third lumbar vertebra level on computed tomography [26–30], there is no objective indicator for older patients who can tolerate highly invasive surgery. Further studies are warranted to identify the risks and benefits for each patient regardless of age.

Minimally invasive surgery, including laparoscopic and robotic-assisted surgery, has been widely accepted in many fields, and a similar trend has been found with respect to liver surgery. Kim et al. revealed that the short- and long-term outcomes of laparoscopic liver surgery for older adult patients were comparable with those of open liver surgery. Additionally, in-hospital days in the laparoscopic liver surgery group were significantly lesser than that in the open liver surgery group [31]. Yoshino et al. demonstrated that surgical outcomes and postoperative complications were comparable between robotic-assisted and laparoscopic major hepatectomy for geriatric patients. Moreover, robotic-assisted hepatectomy showed a lower open conversion rate, shorter length of hospital stay, and shorter intensive care unit stay [32]. Several reports exist on the safety and oncological feasibility of minimally invasive surgery. It is expected that its application for liver surgery will be more widespread in the near future, including in older adults.

The present study had some limitations. First, there is a potential risk of selection bias owing to the retrospective design of the study. Second, the follow-up protocol was not standardized, which could have reduced the power of the TTR data. Third, elderly patients with advanced cirrhosis may have been excluded as surgical candidates preoperatively because of their limited prognoses. Finally, the evaluation of resectability and preselection of suitable candidates for surgery are complex. Even if liver function is not well-preserved, surgery is performed in some cases where the tumor is located near the liver surface. In contrast, if the tumor is located around the hepatic hilum or the root of the hepatic veins, surgeons sometimes hesitate to perform surgery, even in cases of preserved liver function.

## Conclusion

Age alone is not a contraindication for hepatectomy. Hepatectomy, including minor and major hepatectomy, may be safe and oncologically feasible in older patients with HCC who have few comorbidities, good liver function reserve, and good performance status.

### Supplementary Information

Supplementary Material 1.

## Data Availability

No datasets were generated or analysed during the current study.

## Abbreviations

AFP: Alpha-fetoprotein
ALBI: Albumin-bilirubin
ASA-PS: American society of. anesthesiologists physical status
CD: Clavien–Dindo
CI: Confidence interval
CP: Child–Pugh
CSS: Cancer-specific survival
HBV: Hepatitis B virus
HCC: Hepatocellular carcinoma
HCV: Hepatitis C virus
ISGLS: International study group of liver surgery
OS: Overall survival
PVE: Portal vein embolization
TTR: Time to recurrence
