# Supplementary material for: Safety and feasibility of liver resection including major hepatectomy for geriatric patients with hepatocellular carcinoma: a retrospective observational study
Source: BMC Cancer. 2024 Jun 26;24:765. doi: 10.1186/s12885-024-12514-0 (PMC11201318; doi:10.1186/s12885-024-12514-0)
Supplement: Supplementary file 1 — Supplementary Material 1. [file 12885_2024_12514_MOESM1_ESM.pdf]

	Patients < 75 years old (N=593)	%	Patients ≥ 75 years old (N=290)	%	P-value
**Clavien-Dindo**					0.4913
0	406	68.5%	206	71.0%	
1-2	73	12.3%	28	9.7%	
3a-5	114	19.2%	56	19.3%	
Bile leakage	21	3.5%	9	3.1%	
Ascites	8	1.3%	3	1.0%	
Pleural effusion	52	8.7%	30	10.3%	
Abscess	5	0.8%	4	1.4%	
Bleeding	2	0.3%	1	0.3%	
Others	24	4.0%	8	2.7%	
**ISGLS Liver failure**					0.8824
0-A	500	84.3%	248	85.5%	
B-C	77	13.0%	37	12.8%	
Missing	16	2.7%	5	1.7%	
**In-hospital days**	15 (11-19)		14 (11-20)		0.4253

ISGLS: International Study Group of Liver Surgery

Supplementary Table 1. Comparison of postoperative complications, ISGLS liver failure, and in-hospital days between patients aged <75 and ≥75 years in the entire cohort

Clavien-Dindo (≥3a vs. 0-2)	Univariate		Multivariate	
N=883	OR (95%CI)	P-value	OR (95%CI)	P-value
Sex (Male vs. Female)	0.7923 (0.5426-1.1569)	0.2281		
Age (75> vs. ≥75), years old	0.9944 (0.6964-1.1420)	0.9753		
ASA-PS (1-2 vs. 3)	0.9861 (0.5257-1.8497)	0.9652		
ALBI grade (1 vs. 2-3)	0.6589 (0.4708-0.9221)	0.0150*	0.6497 (0.45556-0.9264)	0.0172*
CP score (A vs. B)	1.0390 (0.4208-2.5655)	0.9339		
Operation method (Minor vs. Major)	1.0108 (0.7044-1.4505)	0.9533		
Operation time (300> vs. ≥300), minute	0.3441 (0.2189-0.5409)	<.0001*	0.3311 (0.2012-0.5450)	<.0001*
Estimated blood loss (500> vs. ≥500), mL	0.5647 (0.4031-0.7911)	0.0009*	0.7891 (0.5456-1.1414)	0.2085
Histological fibrosis grade (F0-2 vs. F3-4)	0.6175 (0.4363-0.8742)	0.0066*	0.5858 (0.4096-0.8377)	0.0034*

ISGLS liver failure (B-C vs. 0-A)	Univariate		Multivariate	
N=883	OR (95%CI)	P-value	OR (95%CI)	P-value
Sex (Male vs. Female)	2.1300 (1.2249-3.7039)	0.0074*	1.8828 (1.0631-3.3347)	0.0300*
Age (75> vs. ≥75), years old	1.0322 (0.6778-1.5719)	0.8825		
ASA-PS (1-2 vs. 3)	0.8814 (0.4096-1.8964)	0.7467		
ALBI grade (1 vs. 2-3)	0.4106 (0.2743-0.6146)	<.0001*	0.4359 (0.2856-0.6654)	0.0001*
CP score (A vs. B)	0.4196 (0.2711-0.6493)	<.0001*	0.4083 (0.1775-0.9389)	0.0350*
Operation method (Minor vs. Major)	0.6319 (0.4212-0.9480)	0.0265*	0.7664 (0.4932-1.1910)	0.2369
Operation time (300> vs. ≥300), minute	0.5104 (0.3127-0.8331)	0.0071*	0.7858 (0.4507-1.3702)	0.3955
Estimated blood loss (500> vs. ≥500), mL	0.3592 (0.2395-0.5388)	<.0001*	0.4539 (0.2897-0.7113)	0.0006*
Histological fibrosis grade (F0-2 vs. F3-4)	1.0058 (0.6758-1.4970)	0.9773		

ALBI: albumin-bilirubin, ASA-PS: American Society of. Anesthesiologists physical status, CI: confidence interval, CP: Child-Pugh, ISGLS: International study group of liver surgery, OR: odds ratio

Supplementary Table 2. Univariate and multivariate analyses of postoperative complications and ISGLS liver failure in patients aged <75 and ≥75 years in the entire cohort

	Patients < 75 years old (N=198)	%	Patients ≥ 75 years old (N=78)	%	P-value
**Clavien-Dindo**					0.5916
0	135	68.2%	58	74.4%	
1-2	23	11.6%	7	9.0%	
3a-5	40	20.2%	13	16.7%	
Bile leakage	10	1.7%	4	1.4%	
Ascites	4	0.7%	1	0.3%	
Pleural effusion	15	2.5%	5	1.7%	
Abscess	0	0.0%	1	0.3%	
Bleeding	2	0.3%	0	0.0%	
Others	9	1.5%	2	0.7%	
**ISGLS Liver failure**					0.7532
0-A	161	81.3%	63	80.8%	
B-C	32	16.2%	14	17.9%	
Missing	5	2.5%	1	1.3%	
**In-hospital days**	16 (13-21.3)		15 (13-21)		0.5867

ISGLS: International Study Group of Liver Surgery

Supplementary Table 3. Comparison of postoperative complications, ISGLS liver failure, and in-hospital days between patients aged <75 and ≥75 years in the major hepatectomy cohort

Clavien-Dindo (≥3a vs. 0-2)	Univariate		Multivariate	
N=276	OR (95%CI)	P-value	OR (95%CI)	P-value
Sex (Male vs. Female)	1.0272 (0.4783-2.2060)	0.9451		
Age (75> vs. ≥75), years old	1.2658 (0.6354-2.5216)	0.5026		
ASA-PS (1-2 vs. 3)	0.9479 (0.2578-3.4856)	0.9358		
ALBI grade (1 vs. 2-3)	0.6978 (0.3695-1.3179)	0.2674		
CP score (A vs. B)	not estimated	0.9899		
Operation time (300> vs. ≥300), minute	0.5656 (0.1895-1.6882)	0.3071		
Estimated blood loss (500> vs. ≥500), mL	0.6394 (0.3440-1.1886)	0.1575		
Histological fibrosis grade (F0-2 vs. F3-4)	0.8146 (0.4267-1.5551)	0.5342		

ISGLS liver failure (B-C vs. 0-A)	Univariate		Multivariate	
N=276	OR (95%CI)	P-value	OR (95%CI)	P-value
Sex (Male vs. Female)	2.1774 (0.8151-5.8165)	0.1206		
Age (75> vs. ≥75), years old	0.8944 (0.4547-1.8325)	0.7532		
ASA-PS (1-2 vs. 3)	0.7378 (0.1608-3.3858)	0.6956		
ALBI grade (1 vs. 2-3)	0.6394 (0.3232-1.2650)	0.1989		
CP score (A vs. B)	1.2385 (0.1455-10.539)	0.8448		
Operation time (300> vs. ≥300), minute	0.6667 (0.2221-2.0015)	0.4698		
Estimated blood loss (500> vs. ≥500), mL	0.3791 (0.1867-0.7698)	0.0073*	0.3791 (0.1867-0.7698)	0.0073*
Histological fibrosis grade (F0-2 vs. F3-4)	0.9595 (0.4854-1.8964)	0.9052		

ALBI: albumin-bilirubin, ASA-PS: American Society of. Anesthesiologists physical status, CI: confidence interval, CP: Child-Pugh, ISGLS: International study group of liver surgery, OR: odds ratio

Supplementary Table 4. Univariate and multivariate analyses of postoperative complications and ISGLS liver failure in patients aged <75 and ≥75 years in the major hepatectomy cohort

	Patients < 75 years old (N=593)	%	Patients ≥ 75 years old (N=290)	%	P-value
**Comorbidity**					0.0035*
Cardiac	23	3.9%	30	10.3%	
Pulmonary	11	1.9%	6	2.1%	
Renal	9	1.5%	9	3.1%	
Cardiac & pulmonary	2	0.3%	2	0.7%	
Cardiac & renal	4	0.7%	2	0.7%	
Pulmonary & renal	0	0.0%	1	0.3%	

	Patients < 75 years old (N=198)	%	Patients ≥ 75 years old (N=78)	%	P-value
**Comorbidity**					0.0180*
Cardiac	6	1.0%	9	3.1%	
Pulmonary	3	0.5%	0	0.0%	
Renal	2	0.3%	3	1.0%	
Cardiac & pulmonary	0	0.0%	0	0.0%	
Cardiac & renal	1	0.2%	0	0.0%	
Pulmonary & renal	0	0.0%	0	0.0%	

* indicates that there is a significant difference.

Supplementary Table 5. Comparison of comorbidities between patients aged <75 and ≥75 years in the entire cohort and major hepatectomy cohort
